# The effect of stress ball use during caesarean delivery on maternal anxiety, postoperative pain, and breastfeeding success: a prospective, randomized trial

**DOI:** 10.1590/1806-9282.20260349

**Published:** 2026-08-17

**Authors:** Hatice Selcuk Kusderci, Mesut Oterkuş, Sevda Akdeniz, Cihan Doger, Mehmet Gökhan Taflan, Muharrem Ozbek, Ebru Kayikci, Mustafa Suren

**Affiliations:** 1 Samsun University, Faculty of Medicine, Department of Anesthesiology and Reanimation – Samsun, Türkiye.; 2 Malatya Turgut Özal University, Faculty of Medicine, Department of Anesthesiology and Reanimation – Malatya, Türkiye.; 3 Ankara Bilkent City Hospital, Department of Anesthesiology and Reanimation – Ankara, Türkiye.; 4 Samsun University, Training and Research Hospital, Department of Anesthesiology and Reanimation – Samsun, Türkiye.; 5 Samsun University, Training and Research Hospital – Samsun, Türkiye.

**Keywords:** Stress, Childbirth, Anxiety, Breastfeeding, Non-pharmacological intervention

## Abstract

**OBJECTIVE:**

The aim of this study to evaluate the effects of utilizing a stress ball during cesarean delivery under spinal anesthesia as a simple, non-pharmacological intervention on maternal anxiety, hemodynamic parameters, and lactation outcomes, in order to provide information f77or clinical practice.

**METHODS:**

Cases were randomly separated into two groups. cases in the intervention group were given stress balls and instructed to squeeze them continuously throughout the procedure, from 15 min before spinal anesthesia in the preoperative room until 15 min after newborn delivery. Demographic and surgical data, intraoperative hemodynamic parameters, maternal anxiety and pain levels, lactation adequacy, milk adequacy, and supplemental feeding needs were recorded and analyzed.

**RESULTS:**

Maternal anxiety levels were lower in the stress ball group compared to the control group. Although there was no significant difference in postoperative pain levels, lactation success and perceived milk adequacy were higher in the stress ball group, and supplemental feeding needs were reduced; this highlights the positive effect of the intervention on early lactation outcomes.

**CONCLUSION:**

Although stress ball use did not have a significant effect on pain, we believe that its ability to reduce maternal anxiety may be a supportive, non-pharmacological method that benefits early breastfeeding success.

## INTRODUCTION

In spinal anesthesia, the mother's awareness during the procedure can be a significant source of anxiety. Contributing factors include the duration of the surgery, lack of accurate information or exposure to misinformation about potential complications, and concerns about the baby's health. This anxiety can further increase already elevated stress hormone levels (e.g., cortisol, adrenaline, and noradrenaline) resulting from the surgical procedure, leading to decreased uteroplacental blood flow and thus increased risks to both mother and fetus^1,2^. Furthermore, increased anxiety has been associated with intraoperative hemodynamic instability, increased postoperative pain, delayed breastfeeding, increased risk of respiratory depression from administered sedatives such as midazolam, and weakening or delay of mother-infant bonding^3,4^.

Non-pharmacological interventions such as music therapy, aromatherapy, and breathing exercises have been shown to reduce childbirth-related anxiety effectively^2^. One such method is the use of a stress ball, a form of active distraction involving rhythmic hand squeezing. This technique is believed to modulate pain and anxiety perception through tactile stimulation, thereby creating cognitive engagement by diverting attention away from surgical stressors^5^.

This trial was designed to evaluate the effects of stress ball use during cesarean delivery on perioperative maternal anxiety levels, sedation requirements, postoperative pain, and breastfeeding success.

## METHODS

This research was designed as a prospective, randomized trial. It was between October 2024 and June 2025, involving cases for elective cesarean parturition under spinal anesthesia. Ethical endorsement was obtained from the local institutional review board (Decision No: GOKAEK-2024/16/13, ClinicalTrials no: NCT07073482), and the research was conducted in accordance with the Declaration of Helsinki. All cases were thoroughly informed about the study and provided written informed consent with wet signatures prior to enrollment.

The research contained individuals over 18 years old, classified as The American Society of Anesthesiologists (ASA) physical status II–III, who were undergoing elective cesarean parturition under spinal anesthesia at 37–41 weeks of gestation. Cases were excluded if they had cognitive or psychiatric impairments, underwent emergency caesarean delivery, were using anxiolytic and/or antidepressant medications, had neurological disorders such as diabetes, neuropathies, or required conversion to general anesthesia.

Cases included in the search were randomly separated into two groups using a sealed-envelope method by the independent nurse who was not involved in the search: intervention group (Group S) and control group (Group K).

Intervention group: Patients were instructed to periodically squeeze their umbilical cords from 15 min before spinal anesthesia until 15 min after delivery.

Control group: Standard spinal anesthesia procedure.

Both groups were asked during the procedure, when questioned at 10-min intervals. When questioned at 10-min intervals, cases reporting discomfort were administered a sedative (Dormicum, 0.03 mg/kg, not exceeding 2 mg) in a dose that would not affect their level of consciousness and cognitive function and would not impair their ability to consciously respond to verbal questions. Sedation was administered to both groups after cord clamping. Breastfeeding data were collected within the first 24 h after birth. Milk production was measured using the Test weighing method. In addition to objective criteria such as hypoactivity, blood sugar below 50 mg/dL, and milk weighing, the study investigated infant crying, restlessness, and sleepiness in babies, which can lead mothers to perceive insufficient milk supply. Each pregnant woman received breastfeeding education from the infant nurse.

Cases’ demographic data (age, education level, smoking, employment status, etc.) information, mean arterial pressure (MAP), heart rate, (5th, 10th, 15th, 20th, 25th, 30th, and 35th min), surgical information (surgery duration, anesthesia duration, newborn Apgar score) (1 and 5 min), breastfeeding and complementary feeding, state anxiety (STAI-I) and trait anxiety (STAI-II) (administered 30 min before the operation and repeated 15 min postoperatively) visual analog scale (VAS) for Pain and Anxiety (VAS-A), Patient satisfaction (whether satisfaction increased or decreased due to reduced pain and anxiety, and decreased concerns about infant feeding was investigated) and Sedative Requirement data were recorded in the information form.

### Statistical analysis

The search included 100 cases, divided into two groups of 50 each, with a minimum alpha error of 0.05 and a beta error of 0.8. All analyses were made utilizing Statistical Package for the Social Sciences version 27.0 (IBM Corp., Armonk, NY, USA). A p<0.05 was considered significant. Continuous changeable were compared using independent-samples t-tests or Mann-Whitney U tests, as appropriate. Categorical changeable were analyzed utilizing chi-square tests. Paired samples t-tests were used to assess within-group changes. The normality of continuous change was assessed utilizing the Shapiro-Wilk test. Between-group Comparisons were conducted, making independent samples t-tests for normally dispersion data and Mann-Whitney U tests for non-normally distributed data. Changes in mean arterial pressure over time were analyzed using one way analysis of variance and the Friedman test. Post hoc analyses were made using the Wilcoxon signed-rank test. Heart rate data were normally dispersion; therefore, repeated comparisons were corrected utilizing the Bonferroni adjustment.

For VAS scores and hemodynamic parameters, normality was assessed using the Shapiro-Wilk test. Temporal changes were analyzed utilizing the Friedman test, intergroup comparisons with the Mann-Whitney U test, and pairwise comparisons with the Wilcoxon signed-rank test.

## RESULTS

A total of 108 cases were enrolled in the study, with 53 in Group S and 55 in Group K. The mean age of them was 30.89±4.99 years in Group S and 30.36±5.41 years in Group K, with no significant difference between the groups (p=0.603). The groups were similar in terms of educational status, employment status, Perioperative parameters (including duration of surgery, anesthesia time, and total procedural time), and ASA score (p>0.05). Obstetric history was comparable, with multiparous cesareans the majority in both groups (p=0.996), and the most common parity was the second pregnancy in both groups. Approximately 90% of participants in both groups had a history of previous surgery, with cesarean section being the most common procedure.

A remarkable difference was noted in the use of intraoperative sedation. While no cases in the group S required sedation, 80.0% of cases in the group K did receive sedatives (p<0.001). Regarding neonatal outcomes, 1-min APGAR scores were similar between the groups. However, 5-min APGAR scores were higher in favor of the Group K (p=0.003).

While both situational and trait anxiety scores were similar in the preoperative period, in the postoperative period, Group S exhibited lower anxiety levels than Group K in both situational anxiety (p=0.004) and trait anxiety (p=0.021). According to within-group analyses, Group S showed a significant decrease in both situational and trait anxiety scores after the intervention (p<0.001 for both), whereas Group K showed no significant change.

Postoperative neonatal feeding outcomes were significantly more favorable in Group S. Nearly all mothers in Group S were able to provide sufficient breastfeeding, compared to 65.5% in the control group (p<0.001). Similarly, perceived adequacy of breast milk was higher in Group S (p<0.001). Regarding the need for supplementary feeding, the majority of infants in Group S did not require additional nutrition. In contrast, only 40.0% of infants in Group K were exclusively breastfed (p<0.001) (Table 1).

**Table 1:** Demographic and clinical characteristics, anxiety score, and newborn feeding analysis.

Variable		Group S (n: 53)	Group K (n: 55)	p-value
Age, years		30.89±4.99	30.36±5.41	0.603
Level of education n (%)	Primary school	24 (45.3)	22 (40.0)	0.790
	Middle school	17 (32.1)	21 (38.2)	
	University	12 (22.6)	12 (21.8)	
Working		11 (20.8)	5 (9.1)	0.088
Smoking		8 (15.1)	7 (12.7)	0.722
ASA n (%)	ASA II	53 (100)	54 (98.2)	0.324
	ASA III	0 (0)	1 (1.8)	
Hospitalization history n (%)		48 (90.6)	51 (92.7)	0.685
Obstetric history n (%)	Primipar	6 (11.3)	5 (9.1)	0.996
	Multipar	47 (88.7)	50 (90.9)	
Surgical indication, n (%)	Repeated cesarean section	44 (83.0)	47 (85.5)	0.382
	Breech presentation	4 (7.5)	6 (10.9)	
	Others	5 (9.5)	2 (3.6)	
Perioperative times (min) Median (IQR)	Surgical duration	43 (6)	40 (11)	0.218
	Anesthesia duration	52 (9)	47 (15)	0.156
	Total duration	60.0 (10)	55 (15)	0.089
Sedation is applied n (%)		0 (0)	44 (80)	**<0.001**
Neonatal Apgar score Median (IQR)	1st min	8 (1)	9 (1)	0.430
	5th min	9 (1)	10 (1)	**0.003**
Breastfeeding n (%)	Sufficient	52 (98.1)	36 (65.5)	**<0.001**
	Insufficient	1 (1.9)	19 (34.5)	
Perception of milk adequacy n (%)	Sufficient	52 (98.1)	25 (45.5)	**<0.001**
	Insufficient	1 (1.9)	30 (54.5)	
Additional nutrition n (%)	Yes	6 (11.3)	33 (60)	**<0.001**
	No	47 (88.7)	22 (40)	
STAI-state anxiety (mean±SD)	Preoperative	41.1±3.8	39.8±4.8	0.136
	Posmiddleerative	37.9±3.5	40.3±4.8	**0.004**
	Change (Δ)	-3.19±1.55	-0.46±2.70	
	Intragroup p-value	**<0.001**	0.217	
STAI-trait anxiety (mean±SD)	Preoperative	46.3±4.2	45.2±4.5	0.194
	Posmiddleerative	43.0±3.9	44.9±4.3	**0.021**
	Change (Δ)	-3.28±1.18	0.35±1.57	
	Intragroup p-value	**<0.001**	0.108	

ASA: American Society of Anesthesiologists; IQR: interquartile range; SD: standard deviation. STAI: State anxiety, Δthe difference between postoperative and preoperative STAI scores (ΔSTAI=STAI_post−STAI_pre). Values are given as mean±SD, n (%), or median (minimum–maximum). Chi-square test, the t-test, paired-t-test, and Mann-Whitney U test were used for all categories, p<0.05 was considered statistically significant. Those that are statistically significant are written in bold for emphasis.

No difference in MAP was observed between the groups during the early stages of surgery. However, in the later stages of surgery (30th and 35th min), Group S was higher (p=0.017 and 0.026, respectively). In contrast, heart rate values did not differ between groups at any of the measured time points (Table 2).

**Table 2:** Perioperative mean arterial pressure and heart rate analysis.

	Mean arterial pressure (mmHg)			Heart rate (beats/minute)		
	Group S (n: 53)	Group K (n: 55)	p-value*	Group S (n: 53)	Group K (n: 55)	p-value*
Before spinal	99.3 (91.7–103.7)a,b	95.3 (89.3–100.0)	0.105	98.7±16.5	101.3±17.6	0.429
After spinal	95.3 (85.3–105.0)^b^	96.7 (90.3–101.7)	0.674	95.1±17.2	99.4±18.4	0.214
3rd minute	86.7 (76.7–95.3)a,b	86.0 (69.2–95.2)	0.680	99.7±18.4	98.5±18.7	0.733
5th minute	79.82±15.10	77.96±15.07	0.523	91.8±18.5	97.9±20.4	0.105
10th minute	76.80±16.53	74.97±12.57	0.518	95.7±18.4	98.3±16.1	0.444
15th minute	77.61±13.36	77.69±11.93	0.973	96.1±17.3	97.0±15.6	0.798
20th minute	77.65±11.75	75.03±10.13	0.217	95.7±16.5	97.8±14.3	0.489
25th minute	78.51±10.55	74.92±10.41	0.077	95.1±17.5	99.3±15.0	0.186
30th minute	**79.25±11.13**	**74.24±10.39**	**0.017**	93.9±16.9	98.5±15.1	0.143
35th minute	**80.65±10.29**	**76.16±10.35**	**0.026**	91.9±15.9	96.4±13.9	0.124

Values are given as mean±SD or median (minimum–maximum).

* A t-test or Mann-Whitney U test was used. In mean arterial pressure,

a,b indicates a significant difference compared to a previous time point between those indicated with the same letters, and the Wilcoxon test was used—intragroup assessment of heart rate. Comparisons were made according to baseline values; no significant difference was found at each time point (paired t-test). p<0.05 was considered statistically significant. Those that are statistically significant are written in bold for emphasis.

Postoperative pain assessment revealed a statistically significant difference in favor of Group S at the 9th postoperative hour (p=0.027). VAS-A scores, lower anxiety levels were observed in Group S compared to Group K. Significant differences were noted at the 15th and 35th minutes of the intraoperative period (p=0.011, p=0.021), as well as from the 6th to the 24th postoperative hour, during which Group S consistently exhibited lower VAS-A scores (Table 3).

**Table 3:** Visual analog scale-pain score and visual analog scale-anxiety score analysis.

	VAS-pain score			VAS-anxiety score		
	Group S (n: 53)	Group K (n: 55)	p-value	Group S (n: 53)	Group K (n: 55)	p-value
Preoperative	1.0 (0.0–1.0)	1.0 (0.0–1.0)	0.963	4.0 (3.0–5.0)	5.0 (3.0–6.0)	0.372
Perioperative 15. min	0.0 (0.0–1.0)	0.0 (0.0–1.0)	0.716	**2.0 (1.0–3.0)**	**3.0 (2.0–4.0)**	**0.011**
Recovery duration	0.0 (0.0–1.0)	0.0 (0.0–1.0)	0.855	2.0 (1.0–2.0)	2.0 (1.0–3.0)	0.098
Posmiddleerative 1st hour	1.0 (0.0–3.0)	1.0 (0.0–3.0)	0.700	1.0 (0.0–2.0)	2.0 (1.0–2.0)	0.096
Posmiddleerative 3rd hour	3.0 (2.0–3.0)	2.0 (1.0–4.0)	0.933	1.0 (0.0–1.0)	1.0 (0.0–2.0)	0.055
Posmiddleerative 6th hour	2.0 (2.0–3.0)	3.0 (2.0–3.0)	0.558	**1.0 (0.0–1.0)**	**1.0 (0.0–2.0)**	**0.012**
Posmiddleerative 9th hour	**2.0 (1.0–3.0)**	**2.0 (2.0–3.0)**	**0.027**	**0.0 (0.0–1.0)**	**1.0 (0.0–2.0)**	**0.001**
Posmiddleerative 12th hour	2.0 (1.0–2.0)	2.0 (1.0–3.0)	0.092	**0.0 (0.0–1.0)**	**1.0 (0.0–2.0)**	**0.002**
Posmiddleerative 18th hour	1.0 (1.0–2.0)	2.0 (1.0–2.0)	0.218	**0.0 (0.0–1.0)**	**1.0 (0.0–2.0)**	**0.005**
Posmiddleerative 24th hour	1.0 (0.0–2.0)	1.0 (1.0–2.0)	0.220	**0.0 (0.0–1.0)**	**0.0 (0.0–1.0)**	**0.043**

VAS: visual analog scale, p<0.05 was considered statistically significant. The Mann-Whitney U test was used. Those that are statistically significant are written in bold for emphasis.

## DISCUSSION

In our study, while stress ball use did not lead to a significant difference in intraoperative hemodynamic parameters, it was associated with a decrease in postoperative anxiety, pain levels, and VAS anxiety scores, and an increase in patient satisfaction (reduced pain and anxiety, and a feeling of satisfaction due to reduced concerns about infant feeding). Furthermore, neonatal feeding outcomes were more positive in the intervention group, breastfeeding success increased, the mother's perception of milk adequacy improved, and the need for supplemental feeding decreased.

Preoperative anxiety has been associated with longer recovery time, potentially due to increased anesthetic requirements, and may also contribute to the development of postoperative delirium^6^. Although pharmacological agents are frequently used to manage this anxiety, there has been growing interest in non-pharmacological interventions in recent years. In our study, we employed a stress ball as a simple, non-invasive method of active distraction. The underlying mechanism of such interventions has been discussed in several studies^6-8^. A meta-analysis showed that play-based interventions reduced preoperative anxiety in pediatric patients but did not significantly affect analgesic outcomes^6^. However, the authors acknowledged that the results might be limited by young children's difficulty in verbalizing pain, raising questions about the reliability of pain assessments in this population.

In contrast, Tola et al. demonstrated that non-pharmacological methods such as music therapy and massage, particularly music, effectively reduced both preoperative anxiety and postoperative pain in cases undergoing breast cancer surgery^7^. Similarly, another meta-analysis revealed that music therapy significantly reduced both anxiety and postoperative pain^8^.

In addition to the anxiety triggered by the surgical procedure itself, concerns about the newborn's health further intensify maternal anxiety. Anxiety-induced sympathetic activation and increased oxytocin can contribute to hemodynamic instability, changes in coronary perfusion, increased resistance to uteroplacental blood flow, and preterm birth^9^. These physiological responses can negatively impact both maternal and neonatal outcomes.

The main limitation of pharmacological approaches to managing anxiety during caesarean delivery is the risk of neonatal respiratory depression. In our study, none of the patients in the stress ball group required additional sedative agents, while 80% of the cases in the control group required these agents. Similarly, postoperative anxiety scores were lower in the intervention group. Similarly, postoperative anxiety scores were lower in the intervention group. These findings suggest that the stress ball may be an effective tool in reducing anxiety through tactile distraction and diverting attention away from surgical stimuli. Hunter et al., in their meta-analysis comparing women who had vaginal and caesarean deliveries, demonstrated that music-based interventions significantly reduced anxiety and mild to moderate postoperative pain^10^. Similarly, Simonovich et al. reported that faith-based interventions are widely applicable across various belief systems to effectively reduce maternal anxiety, although their study was conducted among Muslim women^11^.

While heart rate parameters were similar between groups, mean arterial pressure was higher in the intervention group at the 30th and 35th min. However, this elevation was not clinically significant because it remained within normal limits. The lower MAP values in the control group may be attributed to sedative administration.

In our study, although VAS pain scores were lower in the stress ball group at the 9th postoperative hour, no consistent overall difference in pain scores was observed between the groups. While our findings did not demonstrate an apparent analgesic effect of the stress ball, the broader literature suggests that non-pharmacological interventions, particularly those based on music therapy or aromatherapy, may hold promise in reducing postoperative pain in addition to lowering maternal anxiety^7,8^.

Our findings demonstrated that mothers in the stress ball group had higher rates of successful breastfeeding, reported greater milk sufficiency, and their infants had a substantially lower requirement for supplementary feeding compared to the control group. Postpartum hormonal changes play a critical role in lactogenesis; hormones such as prolactin, oxytocin, and cortisol are particularly influential. Among these, oxytocin is a key hormone involved in the milk ejection reflex, acting by stimulating contraction of myoepithelial cells in the mammary glands^12^. Elevated maternal stress is known to suppress oxytocin release, which can delay the initiation of breastfeeding and reduce the volume of milk ejected.

Although maternal stress has not been consistently shown to affect prolactin levels^12^. it may still interfere with milk production through other mechanisms. Previous research suggests that stress may reduce the fat content of breast milk and alter immunologically active components, potentially impacting infant nutrition and immunity^13^.

We think that the stress ball, by lowering maternal anxiety during delivery, may help sustain oxytocin levels, thereby facilitating early milk ejection and improving breastfeeding adequacy. Our findings support this hypothesis, suggesting that even a simple tactile distraction, such as a stress ball, may have clinically meaningful effects on early breastfeeding outcomes and reduce the likelihood of supplemental feeding in the immediate postpartum period.

This search has several limitations. First, it was conducted at a single center, which may limit the generalizability of the findings due to the relatively small sample size and homogenous patient population. Second, because the study focused specifically on anxiety and stress during childbirth, it did not account for psychological factors related to the prenatal or postnatal (puerperal) period. Furthermore, the fact that milk production and MAP may be affected by many factors beyond stress, such as maternal differences, maternal psychology, hormonal factors, pharmacological sedation, and nipple stimulation, depending on the baby's sucking strength, may also limit the study.

## CONCLUSION

Although the study results did not show a direct analgesic effect of stress balls, we consider this non-pharmacological interventions may positively influence both the mother's emotional well-being and newborn feeding. These findings need to be supported by further research.

## Data Availability

The datasets generated and/or analyzed during the current study are available from the corresponding author upon reasonable request.
